# A clock synchronization method based on quantum entanglement

**DOI:** 10.1038/s41598-022-14087-z

**Published:** 2022-06-17

**Authors:** Jianxin Shi, Shanshan Shen

**Affiliations:** 1 Public Foundational Courses Department, Nanjing Vocational University of Industry Technology, Nanjing, 210023 People’s Republic of China; 2 School of Aeronautical Engineering, Nanjing Vocational University of Industry Technology, Nanjing, 210023 People’s Republic of China

**Keywords:** Applied physics, Information theory and computation

## Abstract

This study presents a clock synchronization method based on quantum entanglement which is simple and reliable and more efficient. The clock synchronization method based on quantum entanglement scheme can greatly improve the accuracy and precision of measurement. More importantly, the proposed method avoids synchronization error caused by traditional method due to the asymmetry of two-way delay. The probability of measuring the quantum state oscillates in cosine or sine fashion with propagation delay. The final experimental and simulation results are basically consistent with the theoretical results. The clock synchronization method based on quantum entanglement scheme can greatly imrprove the accuracy and precision of measurement.

## Introduction

As an important physical resource, quantum entanglement has been applied to quantum secure direct communication (QSDC)^1–6^, quantum key distribution (QKD)^7–17^, quantum teleportation (QT)^18,19^, quantum clock synchronization (QCS)^20,21^, renormalization of field theories^22^. It is worth noting that clock synchronization is extremely important in satellite earth quantum communications^23^. This paper relates to the technical field of clock synchronization, especially to a new method of clock synchronization based on quantum entanglement. The methods of clock synchronization is mainly based on two classical protocols proposed by Einstein^24^ and Eddington^25^ respectively. Time synchronization method has been discussed and studied by plenty of researchers in the past two decades, and achievements in various theories and experiments have also been harvested^26–33^. However, in these clock synchronization protocols, actual timing information must be transferred from clock A to clock B through some channels, which generally limit the accuracy of the synchronization. Jozsa et al. firstly proposed the time synchronization protocol implemented by supplying the parties with shared pairs of qubits in known maximally-entangled energy eigenstates^28^. Marko krco et al. extended Jozsa’s protocol to a multiparty version and proposed the ${\text{n}}$-party clock synchronization protocol^29^. Ben-Av et al. have presented an initial entangled state—Z state, which can improve the accuracy of quantum chock synchronization over the straightforwardness of W states^30^. Jingfu Zhang et al. proposed a quantum clock synchronization algorithm in a three-qubit nuclear magnetic resonance (NMR) quantum computer^31^. Xiangyu Kong et al. firstly demonstrated the two multiparty QCS protocols in a four-qubit liquid-state NMR system, and they also proved that Ben-Av’s multiparty QCS algorithm is more accurate than Krco’s^32^. Jie-Dong Yue et al. put forward a quantum clock synchronization scheme of multiple parties, which leverages operation as the trigger to start the evolution of the initial state. They’ve testified that the measurement-triggered quantum clock synchronization is efficient in synchronizing a clock with the average time of other clocks^33^.

At present, the general concept of time synchronization in the industry is basically classical communication, which means that the party with standard time sends the time of its clock to the node to be synchronized, the later then calculates the time differences between its own clock and the standard time and calibrate its own clock^34^. In addition, the accuracy of the time synchronization protocol based on classical physics scheme is scaled as $1/{\sqrt{k}}$, where *k* is the times the protocol is executed. This is known as the Standard Quantum Limit (SQL)^35,36^. Therefore, the accuracy in measuring classic physics scheme is difficult to improve, nor can the methods be guaranteed. We’re in need of a new clock synchronization method with higher efficiency and accuracy.

In this paper, a new clock synchronization method based on quantum entanglement is provided—a new and more efficient quantum clock synchronization method with simplicity and reliability. More importantly, due to the asymmetry of the two-way delay, the method based on quantum entanglement avoids the synchronization error caused by traditional method. The clock synchronization method based on quantum entanglement scheme can greatly imrprove the accuracy and precision of measurement.

This paper is structured as follows: Section “The clock synchronization method based on quantum entanglement” is about the introduction of the new clock synchronization method based on Quantum entanglement. Section “Experiments and simulation results” is about the discussion of the simulation experiment and simulation results. Section “Conclusion” summarizes the entire work and provides some prospects of the method’s e applications.

## The clock synchronization method based on quantum entanglement

Given the above clock synchronization methods, a new clock synchronization method based on quantum entanglement is herein proposed. The method is a one-way time synchronization protocol expected to achieve high-precision clock synchronization through the one-way transmission of qubits. The steps of the method are as follows:

Step1: Adjust the node A and node B to a frequency synchronization state through a time-locked real time clock(RTC) loop which refers to the locking time required for the phase-locked loop to jump from one frequency to another. In this paper, the time-locked loop is driven by RTC whose core component is a crystal oscillator, an integrated circuit, commonly referred to as clock chip. Node A is the party with information about time standard and node B the party to be clocked. With information of time standard, the time of node A is accurate and reliable. The clock standard of Node B needs to move closer to A and be synchronized. The main purpose of Step 1 is to achieve frequency synchronization between node A and node B.

Step 2: Node A prepares a Greenberger-Horne-Zeilinger (GHZ) quantum entangled state at time $t_{1}^{A}$ according to its own clock standard, and sends the quantum entangled state to node B. In Step 2, the quantum entangled state is

1 $$\left| \Psi \right\rangle { = }{{\left( {\left| {000 \cdots 0} \right\rangle { + }\left| {111 \cdots 1} \right\rangle } \right)} \mathord{\left/ {\vphantom {{\left( {\left| {000 \cdots 0} \right\rangle { + }\left| {111 \cdots 1} \right\rangle } \right)} {\sqrt 2 }}} \right. \kern-\nulldelimiterspace} {\sqrt 2 }}$$

where $\left| \Psi \right\rangle$ represents the quantum entangled state which are also called cat state, $\left| {000 \cdots 0} \right\rangle$ and $\left| {111 \cdots 1} \right\rangle$ are qubit streams.

In step 2, after sending the quantum entangled state to node B, node A is also required to send time synchronization information through a classic communication channel, which is usually transmitted by classic protocols, such as bidirectional transmission or loop back transmission. Node B will prioritize the source channel with good communication according to the current clock level of node A included in the time synchronization information. In step 2, the timestamp record at time $t_{1}^{A}$ is the transmission time of photons located at the physical layer and immune from the upper-layer protocol or related device caches.

Step 3: Node B always receives the quantum entangled state under its current clock standard, obtains the density matrix of its own clock standard, and further determines the probability of the first qubit in the standard state. The evolution of the single qubit follows the Schrodinger equation over time. After receiving the quantum entangled state and legally valid time synchronization information, node B will record the time $t_{1}^{B}$ under its clock standard and measure the first qubit with measurement base. The density matrix can be converted to measurement standard, which can be expressed by formula (2).

2 $$\rho^{M} (D) = \frac{1}{2}\left( {\begin{array}{*{20}c} 1 & {e^{in\omega D} } \\ {e^{ - in\omega D} } & 1 \\ \end{array} } \right)$$

where $D$ is the time for free evolution during the transmission of quantum entangled state, $\omega$ is the transition frequency between the energy levels of the two quantum states in the free evolution of the quantum entangled state, and $n$ represents the total number of quantum state energy levels. Time synchronization information can be transmitted through a common channel (or classic channel). As long as the information complies with the relevant provisions of classic channel protocols, the validity of time synchronization information can be judged^37–40^. The probability of first qubit’s standard state can further be obtained by Node B, as shown in formula (3).

3 $$P^{ \pm } = \frac{1}{2}\left[ {1 \pm \cos n\omega D(t_{2}^{B} - t_{1}^{A} - \Delta )} \right]$$

For a relatively accurate probability, multiple measurements are utilized and each one of them obeys the binomial distribution. The probability under the standard state is measured by *N* times, and the average value is taken as the final result. As the finite number of statistics fluctuates, the estimated value of the probability can be uncertain. The standard error is expressed as in the formula (4) .

4 $$\sigma_{P} = \frac{\sigma }{N} = \frac{{\sqrt {NP(1 - P)} }}{N} = \sqrt {{{P(1 - P)} \mathord{\left/ {\vphantom {{P(1 - P)} N}} \right. \kern-\nulldelimiterspace} N}}$$

Among them, $P$ is $P^{ \pm }$, $N$ is the number of measurement statistics.

Step 4: The clock difference $\Delta$ between node A and node B according to the probability $P^{ \pm }$ is calculated. Specifically, the time difference $\Delta$ can be solved according to Eq. (3). Based on the standard error above, the uncertainty of the estimated value of the clock difference can be acquired.

5 $$\delta \Delta \propto \delta D = \frac{{\sqrt {{{P(1 - P)} \mathord{\left/ {\vphantom {{P(1 - P)} N}} \right. \kern-\nulldelimiterspace} N}} }}{{\left| {{{dP} \mathord{\left/ {\vphantom {{dP} {dD}}} \right. \kern-\nulldelimiterspace} {dD}}} \right|}} = \frac{1}{n\omega \sqrt N }$$

Step 5: Node B calibrates its clock standard according to the clock difference Δ, aligns with node A and completes time synchronization^41,42^.

## Experiments and simulation results

A quantum cloud platform Quantum Experience is used for verifying simulation.

**Figure 1:**
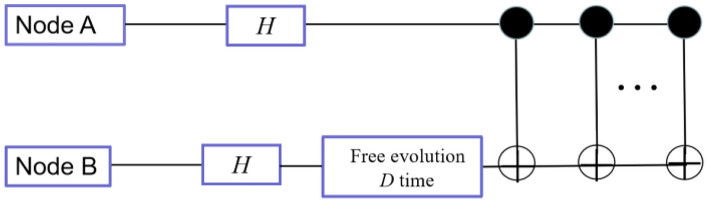
Design idea and procedures of the clock synchronization method based on quantum entanglement.

In the verification of experiment, one Hadamard gate and multiple CNOT gates are used to generate quantum entangled state at node A. The quantum entangled state is allowed to evolve freely for *D* time, the transmission of the qubit in the quantum channel is simulated until it reaches node B. Another Hadamard gate is applied to the first qubit of quantum entangled state and measured the output result so as to simulate the measurement process of node B by means of the measurement basis in step 3 (Fig. 1).

**Figure 2:**
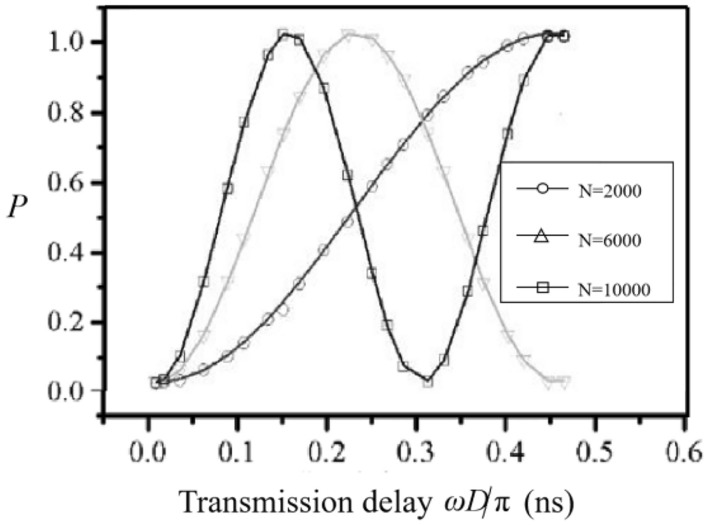
The theoretical and experimental results of the clock synchronization method based on quantum entanglement.

The theoretical and experimental results are shown in Fig. 2. Abscissa represents delay in nanosecond transmission, ordinate represents the probability of measuring the quantum state. In order to lower the uncertainty in the results of quantum measurement, Fig. 2 demonstrates the final effect when different values of *N* are taken in the experiments. The smooth curve reflects the theoretical effect and the shape line on it represents simulation effect. The circle shape represents the effect when *N* is set as 2000, the triangle shape represents the effect when* N* is set as 6000, and the square shape represents the effect when *N* is set to 10,000 times. The probability of measuring the quantum state oscillates in a cosine or sine manner with propagation delay. It can be seen from Fig. 2 that the final experimental outcomes are basically consistent with the theoretical results. Besides from that, the uncertainty of the estimated value of the clock difference is related to the energy level of quantum state and measurement statistic. In the experiments, the uncertainty of clock difference estimates can be minimized to 10^−4^, and the synchronization error caused by the asymmetry of the two-way delay of the traditional method can also be avoided when the energy level *n* of the quantum state and the measurement statistics is selected properly.

**Figure 3:**
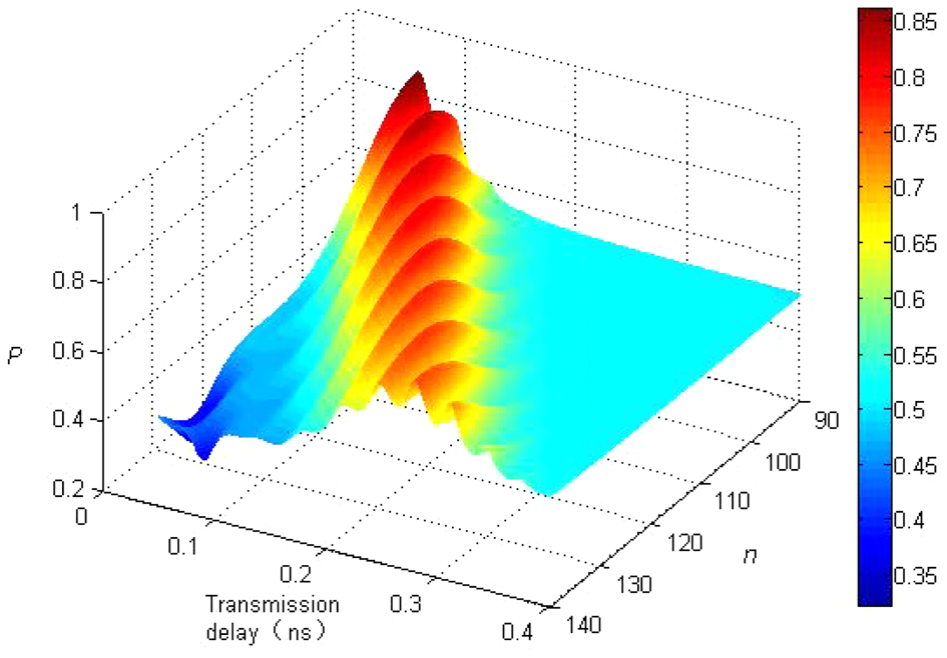
The simulation results of clock synchronization method when taking the impact of decoherence factors into account.

The above experiments only take account of the phase evolution of qubits. The probability of measuring quantum state oscillates periodically in a cosine or sine fashion with propagation delay, and the amplitude of the oscillation does not taper with it. As we all know, the impact of other factors, such as the noises of vacuum fluctuation, Raman scattering and other nonlinear effects should also be taken into consideration, they may affect the transmission process and results of clock synchronization method based on quantum entanglement, therefore, calculation results of the interference effects of these factors in the following simulation experiments are shown in Fig. 3.

Figure 3 is displayed with simulation results with the influence of decoherence factors considered, and the measurement statistic *N* is set to 6000, which corresponds to the triangular shape data in Fig. 2. In Fig. 3, the abscissa represents the transmission delay in nanoseconds, the ordinate represents the energy level of the quantum state, and the Z-axis represents the probability of measuring the quantum state. If the influence of vacuum fluctuation noises, Raman scattering and other nonlinear effects is taken into account, it’s clear in the Fig. 3 that a series of peaks rise with propagation delay of approximately 0.3 ns, which is consistent with the conclusion of *N* = 6000 shown in Fig. 2.

In addition, through simulation experiments, it is further found that when the frequency of measurements *N* is fixed, errors in measurement can be lowered when the number of quantum energy levels is increased. In terms of P ≈ 0.3, *N* = 6000, the standard error according to Eq. (4) can be as low as 5.92 × 10^−3^. The energy level *n* of quantum state is set between 90 to 140 in compliance with the Eq. (5), and the smallest uncertainty of clock difference we can estimate is about 1.8 × 10^−4^. It’s obvious from Fig. 3 that the experimental and simulation results basically resonate with the theoretical results. In reference^31–33^, due to the limitation of NMR control system, the experimental accuracy they demonstrate is on the order of microseconds, while our experimental accuracy is on the order of nanoseconds. The clock synchronization method based on quantum entanglement scheme can greatly imrprove the accuracy and precision of measurement. These results will contribute to the research of entanglement-based secure quantum cryptography and quantum communication.

## Conclusion

In conclusion, this paper provides a clock synchronization method based on quantum entanglement with the capacity of greatly improving the accuracy and precision of measurement. The fact that the synchronization error caused by the asymmetry of the two-way delay of caused by the traditional method is avoided proves that experimental and simulation results are basically consistent with the theoretical results. The clock synchronization method based on quantum entanglement will contribute to the research of entanglement-based secure quantum cryptography and quantum communication.
